# The relationship between cancer patients’ spiritual needs, quality of life and depression levels: A correlational study

**DOI:** 10.1017/S1478951525100989

**Published:** 2025-11-07

**Authors:** Hakime Aslan, Hanife Çelik, Behice Erci

**Affiliations:** 1 Department of Fundamentals of Nursing, Faculty of Nursing, Inonu University, Malatya, Türkiye; 2 Disabled Care and Rehabilitation Program, Vocational School of Health Services, Bingol University, Bingol, Türkiye; 3 Department of Public Health Nursing, Faculty of Nursing, Inonu University, Malatya, Türkiye

**Keywords:** Depression, patient, cancer, spiritual needs, quality of life

## Abstract

**Objectives:**

This study was conducted to examine the relationship between cancer patients’ spiritual needs and their quality of life and depression levels.

**Methods:**

This cross-sectional, exploratory study was conducted between March 2023 and November 2024. The study population consisted of cancer patients hospitalized in medical oncology departments at a university hospital in eastern Turkey. The sample consisted of 250 patients, determined by power analysis. To collect data, the “Demographic Information Form,” “Spiritual Needs Assessment Scale,” “EORTC QLQ-C30 Version 3.0 Quality of Life Scale,” and “Beck Depression Scale” were used to evaluate the patients’ sociodemographic characteristics and disease process.

**Results:**

There was a weak, negative, statistically significant relationship between patients’ spiritual needs and the subdimensions of the quality of life scale, specifically the general perceived health status (*r* = −0.297, *p* < 0.001), physical (*r* = −0.446, *p* < 0.001), role (*r* = −0.423, *p* < 0.001), emotional (*r* = −0.472, *p* < 0.001), cognitive (*r* = −0.458, *p* < 0.001) and social (*r* = −0.443, *p* < 0.001) functions, and finally, a weak positive correlation was found between the symptoms experienced (*r* = 0.376, *p* < 0.001) and depression levels. Additionally, a weak positive correlation between spiritual needs and depression level (*r* = 0.374, *p* < 0.001) was identified. Functional areas, depression, education level, diagnosis duration, and symptoms were identified as variables predicting spiritual needs.

**Significance of results:**

In conclusion, it was determined that as the spiritual needs of cancer patients increased, their quality of life decreased and the severity of depression increased.

## Introduction

Life-threatening diseases such as cancer increase a person’s physical, psychological, and spiritual needs due to the nature of the disease, its long-term course, and the frequency of symptoms experienced (Kang and Choi 2020; McClement and Chochinov 2010). Spiritual needs are multidimensional, comprehensive, and personal, and are also related to an individual’s religious and cultural background. Spiritual needs can be structured into four dimensions: existential needs, religious needs, inner peace needs, and giving/productivity and forgiveness needs (Büssing et al. 2010; Kang and Choi 2020). Cancer patients whose spiritual needs are met are better able to cope with the physical and mental distress and consequences of cancer treatment (Riba et al. 2019). In a study conducted in the literature, it was determined that almost all (94%) of cancer patients reported at least one spiritual need, with the most important needs being inner peace and active/productive forgiveness (Höcker et al. 2014). Another study identified the important spiritual needs of cancer patients as the need for wholeness and security, the need for social support, the need for forgiveness and the preservation of inner peace, and the opportunity to talk about anxiety and sadness (Haußmann et al. 2017).

Cancer patients’ high spiritual needs seriously affect their quality of life (Riba et al., 2019). Quality of life is a multidimensional concept. It reflects individuals’ perceptions of their physical, psychological, social, and environmental conditions (Felce and Perry 1995). Studies conducted on cancer patients have shown a positive relationship between the satisfaction cancer patients feel due to their spiritual needs being met and their quality of life (Bai and Lazenby 2015). Similarly, a positive relationship has been found between spiritual well-being and quality of life in cancer patients, and it has been noted that spiritual well-being has protective effects against end-of-life despair (Johnson et al. 2007). Willemse and colleagues (Willemse et al. 2020) noted that spirituality is a fundamental component of quality of life and demonstrated that effective spiritual care alleviates the distress of patients and their loved ones (Willemse et al. 2020). Additionally, studies have shown that patients with high spiritual well-being also have high quality of life (Koenig, 2020; VanderWeele 2017; Belk and Balboni, 2024). This finding suggests that spiritual support approaches can improve quality of life in cancer patients by influencing it.

The diagnosis and treatment processes, symptoms, anxiety, and fear experienced by cancer patients cause significant changes in their quality of life and increase the risk of psychological problems such as depression (Walker et al. 2014). Depression is a common mental health problem in cancer patients and is an important factor that negatively affects quality of life (Hohls et al. 2021). It is known that depression reduces patients’ adherence to treatment and makes the disease process more difficult (DiMatteo and Haskard-Zolnierek 2011). However, spiritual support and psychosocial interventions have been shown to be effective in reducing depression (Caminiti et al. 2021; Reisi et al. 2021). In a study by Ramezani et al., it was determined that unmet spiritual needs were high among elderly individuals with chronic illnesses, and that depression and disease duration were factors affecting the spiritual needs of elderly individuals (Ramezani and Karimi 2019). Similarly, spiritual care, a fundamental aspect of supportive care for cancer patients, has been found to reduce the frequency of anxiety and depression and improve quality of life (Whitford et al., 2008). In this context, it can be said that meeting spiritual needs has the potential to improve quality of life by reducing depression levels.

This study aims to conduct a comprehensive evaluation of the relationship between the spiritual needs of cancer patients and their quality of life and depression levels.

Cancer patients’ experiences of illness, diagnosis, treatment, symptoms, anxiety, and fear increase their risk of developing psychological problems (Walker et al. 2014). Depression is a common psychological problem that significantly affects the quality of life of cancer patients (Hohls et al. 2021). Depression is a condition that reduces patients’ adherence to treatment and complicates the process (DiMatteo and Haskard-Zolnierek 2011). However, the literature has found that spiritual support and psychosocial interventions are effective in reducing depression in patients (Caminiti et al. 2021; Reisi et al. 2021). A study by Ramezani and colleagues found that individuals with chronic illnesses have high levels of unmet spiritual needs and that depression and illness duration are factors that affect individuals’ spiritual needs (Ramezani and Karimi 2019). Similarly, spiritual care, which is a fundamental component of supportive care for cancer patients, has been found to reduce the incidence of anxiety and depression and improve quality of life in a positive way (Whitford et al., 2008). In this context, it can be stated that meeting spiritual needs has the potential to improve quality of life by reducing depression levels in individuals.

This study was conducted to thoroughly investigate and evaluate the relationship between the spiritual needs of cancer patients and their quality of life and depression levels.

## Methods

**Type of research**: This study is a cross-sectional and correlational type. The research was conducted between March 2023 and November 2024.

**Population-sample**: The population of the research consisted of cancer patients hospitalized in medical oncology departments at a university hospital in eastern Turkey. The sample consisted of 240 patients determined by power analysis (power analysis with a 0.05 error rate, a 0.95 confidence interval, and 90% representation power). Data collection continued until the sample size determined during the research process was reached. The study was concluded with 250 patients. Convenience sampling was used to select the sample from the population. Data were collected from patients who were hospitalized and met the inclusion criteria for the study.

### Inclusion criteria

All patients who were conscious, had no severe physical symptoms, were able to communicate, had been diagnosed with cancer for at least 6 months, agreed to participate in the study, and were over 18 years of age were included in the study.

### Exclusion criteria

Patients in the terminal stage, with severe respiratory failure (requiring continuous oxygen use), or with visual and/or auditory impairments were excluded from the study. Additionally, patients who completed the data collection forms incompletely or incorrectly, or who wished to withdraw from the study, were excluded.

### Data collection

The data were collected by the researcher using face-to-face interviews. The researcher visited the hospital, provided the necessary explanations about the study in the patients’ rooms, and obtained verbal or written consent from the patients. The questionnaires were given to the patients and they were given the necessary time (10–15 minutes) to fill them out. Four separate data collection forms were used as data collection tools in the study. The researcher used the “Demographic Information Form,” the “Spiritual Needs Assessment Scale,” the “EORTC QLQ-C30 Version 3.0 Quality of Life Scale,” and the “Beck Depression Scale” to assess the patients’ sociodemographic characteristics and disease process.

### Spiritual needs assessment scale of patients with cancer (SNASPC)

The scale developed by Hatamipour and colleagues aims to measure the spiritual needs of cancer patients (Hatamipour et al. 2018). The scale consists of 5 subscales and 38 items. The 7-point Likert-type scale can yield scores ranging from 38 to 266, with higher scores indicating higher spiritual needs in cancer patients. The Turkish validity and reliability of the scale were assessed by Erci and Aslan in 2022, and the Cronbach’s α value of the scale was found to be 0.85 (Erci and Aslan 2022). In this study, the Cronbach’s α value of the scale was determined to be 0.90.

### EORTC quality of life scale (EORTC QLQ-C30)

Developed by the European Organisation for Research and Treatment of Cancer (EORTC) (2001), the EORTC QLQ-C30 Version 3.0 quality of life scale is a widely used measure in cancer patients worldwide. The scale consists of 30 questions. Scores range from 0 to 100. High scores in the general well-being and functional domains indicate a high quality of life. Low scores on the symptoms section indicate high quality of life. This self-administered questionnaire incorporates five functional scales [physical (PF), role (RF), cognitive (CF), emotional (EF), and social (SF) functioning scales] and three symptom scales (fatigue, pain, and nausea/vomiting), a global health/ QOL scale, and several single items for the assessment of additional symptoms commonly reported by cancer patients (e.g. dyspnea, loss of appetite, sleep disturbance, constipation, and diarrhea), as well as the perceived financial impact of the disease and treatment. All items are scored on 4-point Likert scales, ranging from 1 (not at all) to 4 (very much). As an exception, two items (items 29 and 30) in the global health/QOL subscale were scored on a modified 7-point linear analogue scale (Fayers et al. 2001). The Turkish validity and reliability study of the scale was conducted by Cankurtaran et al. in 2008 and found that Cronbach’s Alpha coefficient ranged from 0.56 to 0.85 in the subscales (Cankurtaran et al. 2008). In this study, Cronbach’s Alpha coefficient was found to be 0.85.

### Beck depression scale (BDS)

Developed in 1961 by Beck et al. to measure behavioural symptoms of depression in adolescents and adults (Beck 1961), the entire scale was revised in 1978 to remove duplicates that defined severity, and patients were asked to evaluate their condition over the past week, including the present day. Severity is interpreted as follows: 0–9 = Minimal, 10–16 = Mild, 17–29 = Moderate, 30–63 = Severe. The validity and reliability analysis of the scale in Turkish was conducted in 1989 (Hisli 1989). In this study, Cronbach’s Alpha coefficient was found to be 0.85.

### Data analysis

The data were analyzed using the SPSS 28.0 software package. The normality of the data distribution was analyzed using the Kolmogorov–Smirnov and Shapiro–Wilk tests. The data were found to be normally distributed (*p* > 0.05). In the statistical analysis of the study, descriptive statistics, mean, standard deviation, Student’s *t*-test, one-way ANOVA, Pearson correlation tests, and linear regression analysis were used. Statistical significance was accepted as *p* < 0.05.

### Ethical dimension of the study

Ethical approval for the study was obtained from the Scientific Research and Publication Ethics Committee of X University (E-33117789-044-66092-22/13; Decision No. 2). In addition, written institutional permission was obtained from the hospital directorate. Patients were informed about the study, and written or verbal consent was obtained from those who agreed to participate.

## Results

Information regarding the sociodemographic variables of the patients is provided in Table 1. The mean age of the patients participating in the study was 55.28 ± 13.95 years; they had been receiving treatment for an average of 2.97 ± 1.23 years and had received an average of 9.17 ± 2.63 courses of chemotherapy treatment. It was determined that 49.6% of the patients were female, 67.2% were married, 27.6% were university graduates, 48.4% perceived their income level as good, and 53.2% were unemployed. According to disease variables, 19.6% of patients were diagnosed with stomach cancer, 33.6% were in stage IV of the disease, 35.2% received chemotherapy and surgical treatment, and 44.4% underwent surgery due to their disease (Table 1).

**Table 1.** Sociodemographic information of the patients

Sociodemographic information (*N* = 250)		X ± SD	
		55.28 ± 13.95	
Age		*n*	%
Gender	Female	124	49.6
	Male	126	50.4
Marital status	Married	168	67.2
	Single	82	32.8
Education level	Illiterate	33	13.2
	Literate	74	29.6
	Primary education	44	17.6
	High School	30	12
	University and above	69	27.6
	High	125	48.4
Perceived economic level	Middle	89	34.5
	Low	36	14
Working status	Working	83	33.2
	Not working	133	53.2
	On leave	34	13.6
**Diagnosis status and treatment process**			
Diagnosis	Breast CA	42	16.8
	Lung CA	41	16.4
	Stomach CA	49	19.6
	Colorectal CA	32	12.8
	Uterus CA	29	11.6
	Other	57	22.1
Stage of disease	I	42	16.8
	II	57	22.8
	III	67	26.8
	IV	84	33.6
Treatment	Chemotherapy	9	3.6
	Radiotherapy	25	10
	Chemotherapy + radiotherapy	82	32.8
	Chemotherapy + surgery	88	35.2
	Chemotherapy + radiotherapy + surgery	46	18.4
Have you had surgery for the disease?	Yes	111	44.4
	No	139	55.6
		X ± SD	
**Diagnoses time (years)**		2.97 ± 1.23	
**Number of chemotherapy cure**		9.17 ± 2.63	

X: mean, SD: standard deviation.

Cancer patients scored a total of 201.98 ± 33.4 points on the spiritual needs assessment scale, indicating that their spiritual needs were high. They scored a total of 53.52 ± 7.09 points on the quality of life scale, indicating that their quality of life was low. In particular, it was observed that the symptoms experienced (67.36 ± 27.02) were the most important factor reducing quality of life. Patients scored 41.29 ± 14.32 points on the Beck Depression Scale, and it was determined that their depression levels were severe (Table 2).

**Table 2.** Spiritual needs assessment scale of cancer patients, EORTC quality of life scale and beck depression scale mean points

Scale	**Min**−**Max**	X ± SD
**Spiritual needs assessment scale of patients with cancer-(SNASPC) total**	95.0−266.0	**201.98 ± 33.4**
Religious need	9.0−63.0	47.69 ± 11.05
Finding meaning and purpose	7.0−49.0	37.20 ± 8.89
Seeking peace	19.0−70.0	53.25 ± 9.67
The need to communicate	6.0−42.0	32,04 ± 7.28
Support and independence	6.0−42.0	31,83 ± 7.29
**EORTC quality of life scale (EORTC QLQ-C30)**		**53.52 ± 7.09**
**Global quality of life (QOL)**	0.0−100.0	**60.50 ± 21.07**
**Functional scales**	0.0−100.0	**32.72 ± 24.34**
Physical functioning (PF)	0.0−100.0	33.46 ± 20.09
Role functioning (RF)	0.0−100.0	32.66 ± 25.33
Emotional functioning (EF)	0.0 − 100.0	32.23 ± 24.57
Cognitive functioning (CF)	0.0−100.0	33.03 ± 25.78
Social functioning (SF)	0.0−100.0	32.26 ± 25.98
**Symptom scales**		**67.36 ± 27.02**
Fatigue (FA)	0.0−100.0	66.44 ± 27.44
Nausea and vomiting (NV)	0.0−100.0	69.04 ± 28.57
Pain (PA)	0.0−100.0	67.33 ± 27.81
Dyspnea (D)	0.0−100.0	66.00 ± 28.33
Sleep disturbance (SD)	0.0−100.0	65.06 ± 29.23
Appetite loss (AL)	0.0−100.0	66.93 ± 29.11
Constipation (C)	0.0−100.0	69.06 ± 26.62
Diarrhea (DI)	0.0−100.0	67.86 ± 29.77
Financial impact (FI)	0.0−100.0	68.53 ± 29.21
**Beck depression scale (BDS)**	2.00−63.00	**41.29 ± 14.32**

X: mean, SD: standard deviation.

According to the results of the correlation analysis, there was a weak negative correlation between patients’ spiritual needs and the subscales of the quality of life scale: general perceived health status (*r* = −0.297), physical (*r* = −0.446), role (*r* = −0.423), emotional (*r* = −0.472), cognitive (*r* = −0.458), and social (*r* = −0.443) functions, and finally, a weak negative correlation with symptoms (*r* = 0.376). An increase in spiritual needs was associated with a negative decrease in perceived general health status and functional areas, which are positive indicators of quality of life, while it was positively associated with symptoms. The positive relationship identified between spiritual needs and experienced symptoms indicates that the needs in both areas are closely related.

Additionally, a weak but statistically significant positive relationship was found between spiritual needs and depression levels (*r* = −0.374). It was determined that as patients’ spiritual needs increased, the severity of depression also increased (Table 3).

**Table 3.** Relationship between cancer patients’ spiritual needs, quality of life, and depression levels

		1	2	3	4	5	6	7	8	9
Spiritual Needs Assessment Scale of Patients with Cancer-(SNASPC) Total **(1)**	*r*	1								
	*p*									
Global quality of life (QOL) **(2)**	*r*	−0.297**	1							
	*p*	0.001								
Physical functioning (PF) **(3)**	*r*	−0.446**	−0.037	1						
	*p*	0.000	0.562							
Role functioning (RF) **(4)**	*r*	−0.423**	−0.035	0.906**	1					
	*p*	0.000	0.579	0.000						
Emotional functioning (EF) **(5)**	*r*	−0.472**	−0.038	0.948**	0.914**	1				
	*p*	0.000	0.553	0.000	0.000					
Cognitive functioning (CF) **(6)**	*r*	−0.458**	−0.016	0.938**	0.882**	0.957**	1			
	*p*	0.000	0.798	0.000	0.000	0.000				
Social functioning (SF) **(7)**	*r*	−0.443**	−0.042	0.928**	0.899**	0.965**	0.946**	1		
	*p*	0.000	0.510	0.000	0.000	0.000	0.000			
Symptom scales total score **(8)**	*r*	0.376**	0.022	−0.963**	−0.922**	−0.973**	−0.956**	−0.961**	1	
	*p*	0.000	0.730	0.000	0.000	0.000	,000	0.000		
Beck depression scale (BDS) **(9)**	*r*	0.374**	0.058	−0.187**	−0.187**	−0.180**	−0.193**	0.192**	0.198**	1
	*p*	0.000	0.359	0.003	0.003	0.004	0.002	0.002	0.002	

** Correlation is significant at the 0.01 level (2-tailed).

The differences between the sociodemographic and disease characteristics of cancer patients and their spiritual needs are presented in Table 4. No significant differences were found between gender, marital status, perceived income level, employment status, and spiritual needs (*p* > 0.05). However, a statistically significant difference was found between educational level and spiritual needs scale score (*p* < 0.05), with patients with a university education or higher having higher levels of spiritual needs. Among disease characteristics, disease stage was found to be a variable affecting spiritual needs (*p* < 0.05), with cancer patients at stage IV having higher spiritual needs. No significant difference was found between disease diagnosis, treatment type, and surgical status and the average spiritual needs scale score (*p* > 0.05).

**Table 4.** Comparison of patients’ sociodemographic and disease characteristics with spiritual needs score averages

Variables		Spiritual Needs Assessment Scale of Patients with Cancer-(SNASPC) X ± SD
Gender	Female	204.38 ± 34.00
	Male	202.60 ± 33.05
	Test (*t*)	3.656
	Significance (*p*)	0.552
Marital status	Married	199.89 ± 30.82
	Single	204.00 ± 34.76
	Test (*t*)	1.090
	Significance (*p*)	0.491
Education Level	Illiterate	204.72 ± 37.00
	Literate	201.87 ± 35.51
	Primary education	200.50 ± 31.45
	High School	199.60 ± 38.13
	University and above 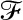	217.17 ± 26.14
	Test (*F*)	3.801
	Significance (*p*)	0.001**
Perceived economic level	High	201.56 ± 32.52
	Middle	203.25 ± 32.57
	Low	204.30 ± 39.36
	Test (*F*)	1.062
	Significance (*p*)	0.444
Working status	Working	203.80 ± 33.71
	Not working	208.50 ± 34.39
	On leave	205.60 ± 27.62
	Test (KW)	4.125
	Significance (*p*)	0.322
**Diagnosis status and treatment process**		
Diagnosis	Breast CA	200.14 ± 34.45
	Lung CA	202.26 ± 35.47
	Stomach CA	199.65 ± 32.15
	Colorectal CA	198.25 ± 38.09
	Uterus CA	205.93 ± 27.10
	Other	207.38 ± 30.70
	Test (KW)	2.955
	Significance (*p*)	0.108
Stage of Disease	I	200.69 ± 30.44
	II	206.15 ± 28.87
	III	204.47 ± 28.92
	IV 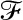	211.28 ± 30.51
	Test (*F*)	2.394
	Significance (*p*)	0.030*
Treatment	Chemotherapy	192.11 ± 31.64
	Radiotherapy	198.52 ± 38.72
	Chemotherapy + radiotherapy	207.41 ± 29.02
	Chemotherapy + surgery	199.80 ± 34.11
	Chemotherapy + radiotherapy + surgery	207.93 ± 33.49
	Test (KW)	7.148
	Significance (*p*)	0.128
Have you had surgery for the disease?	Yes	205.51 ± 32.09
	No	202.35 ± 34.67
	Test (*t*)	−1.194
	Significance (*p*)	0.405
Age		*r* = 0.192*
		***p* = 0.030**
Diagnoses time (years)		*r* = 0.258**
		***p* = 0.000**
Number of chemotherapy cure		*r* = − 0.059
		*p* = 0.349

* *p* < 0.05;

** *p* < 0.001, X: mean, SD: standard deviation, *t*: independent sample *t*-test, *F*: Oneway ANOVA, KW: Kruskal–Wallis Test, *r*: correlation.

There was a weak but statistically significant positive correlation between age and duration of diagnosis and spiritual needs (*p* < 0.05). As age and duration of diagnosis increased, spiritual needs also increased. However, there was no significant correlation between the duration of chemotherapy and spiritual needs (Table 4).

Linear regression analysis stepwise model was used to determine the factors affecting patients’ spiritual needs. The independent variables included age, education level, disease stage, and diagnoses time (years), which were found to be significant in the initial analysis, as well as the perceived general health status, functional domain, and symptoms subdomains of the QLQ-C30 quality of life scale, and the total score of the Beck Depression Scale. The total score of the Spiritual Needs Assessment Scale for Cancer Patients was used as the dependent variable. According to the regression analysis results, the functional domain of the QLQ-C30 quality of life scale had an effect size of 0.19, the functional domain and Beck Depression Scale total had an effect size of 0.23, the functional domain, Beck Depression Scale, and diagnoses time had an effect size of 0.24, the functional domain, Beck Depression Scale, time since diagnosis, and educational level had an effect size of 0.25, and the functional domain, Beck Depression Scale, duration since diagnosis, educational level, and QLQ-C30 quality of life scale symptom subdomain had an effect size of 0.26 on the spiritual needs of cancer patients (*p* < 0.001). The effect of qualitative data-related characteristics on the spiritual needs of cancer patients was determined and found to be *R* = 0.515, *R*^2^ = 0.264, and it was statistically significant (*p* < 0.001) that 26.4% of the total variance in the dependent variable of the cancer patients’ spiritual needs assessment scale was explained by these variables. The functional domain of the quality of life scale was found to have the greatest independent effect on spiritual needs. In the fifth model, age, disease stage, and the perceived general health subdomain of the QLQ-C30 quality of life scale were excluded from the model because they did not affect spiritual needs (Table 5).

**Table 5.** Regression analysis of the effect of quality of life, depression, and demographic variables on spiritual needs

	Unstandardized coefficients		Standardized coefficients						
Model	*β*	SE	*β*	*t*	Sig.	*F*	Sig	*R*	Adjusted *R^2^*
**1(Constant)**	217.109	5.069		70.735	0.000				
QLQ-C30 Functional scales Total	−0.462	0.072	−0.437	−6.416	0.000	51.163	0.000^b^	0.437^a^	0.192
**2(Constant)**	184.469	6.584		48.020	0.000				
QLQ-C30 Functional scales Total	−0.388	0.069	−0.317	−6.409	0.000	44.283	0.000^c^	0.486^b^	0.230
Beck Depression Scale Total	0.732	0.132	0.313	5.523	0.000				
**3(Constant)**	194.382	7.823		34.848	0.000				
QLQ-C30 Functional scales Total	−0.375	0.069	−0.316	−6.438	0.000	32.653	0.000^d^	0.504^c^	0.244
Beck Depression Scale Total	0.731	0.131	0.313	5.564	0.000				
Diagnoses time (years)	3.456	1.503	0.127	2.299	0.022				
**4(Constant)**	184.800	9.097		32.314	000				
QLQ-C30 Functional scales Total	−0.375	0.069	−0.315	−6.473	0.000	28.727	0.000^e^	0.515^d^	0.253
Beck Depression Scale Total	0.743	0.131	0.318	5.687	0.000				
Diagnoses time (years)	3.146	1.502	0.116	2.095	0.037				
Education Level	2.618	1.291	0.112	2.028	0.044				
**5(Constant)**	176.522	9.828		24.961	0.000				
QLQ-C30 Functional scales Total	−0.374	0.017	−0.315	−6.486	0.000	24.86	0.000^f^	0.528^e^	0.264
Beck Depression Scale Total	0.752	0.130	0.322	5.796	0.000				
Diagnoses time (years)	3.249	1.502	0.120	2.178	0.030				
Education Level	2.754	1.283	0.117	2.144	0.033				
Symptom scales Total	2.181	1.021	0.116	2.136	0.034				

Dependent Variable: Spiritual Needs Assessment Scale of Patients with Cancer-(SNASPC).

**aPredictors:** (Constant), QLQ-C30 Functional Scales Total.

**bPredictors:** (Constant), QLQ-C30 Functional Scales Total, Beck Depression Scale Total.

**cPredictors:** (Constant), QLQ-C30 Functional Scales Total, Beck Depression Scale Total, Diagnoses time (years).

**dPredictors:** (Constant), QLQ-C30 Functional sSales Total, Beck Depression Scale Total, Diagnoses time (years), Education Level.

**ePredictors:** (Constant), QLQ-C30 Functional Scales Total, Beck Depression Scale Total, Diagnoses time (years), Education Level, Symptom scales Total.

f ANOWA test

Although the initial analysis revealed significant differences between age, disease stage and spiritual needs, regression analysis showed that these factors were not effective (Table 5).

## Discussion

This study examined the relationship between cancer patients’ spiritual needs and their quality of life and depression levels, and assessed the variables affecting spiritual needs in a multidimensional manner.

The results of the study showed that cancer patients had high spiritual needs and depression levels, while their quality of life was low. The average spiritual needs assessment scale score of cancer patients was 201.98 ± 33.4, indicating that their spiritual needs were high, with particularly high needs in the subdimensions of “search for peace” and “religious needs.” Similar research results have been reported in the literature. A study conducted on Indonesian Muslim cancer patients indicated that the patients’ spiritual needs were high, with religious needs being particularly prominent (Sastra et al. 2021). In a study conducted with advanced cancer patients, it was found that general spiritual needs were high and closely related to prolonged disease course, repeated hospitalizations, and deterioration of the patient’s physical condition (Shi et al. 2023). Another study with cancer patients found that patients often turn to their religion/spirituality to find meaning when faced with illness (Zumstein-Shaha et al. 2020). Wisesrith et al. also found that the spiritual needs of terminally ill cancer patients were of moderate level; the highest average spiritual needs of patients were found to be, in order, “preparing for death,” “having meaning, values, and life goals,” and “having the opportunity to pursue the most important things in life” (Wisesrith et al. 2021). This research result supports the literature. In diseases with a risk of death, such as cancer, questioning the meaning of life, finding meaning and purpose, and seeking refuge in religion and spirituality to cope are very common practices among Muslim patients (Erci and Aslan 2022). Meeting the increasing needs in these areas is a crucial factor in improving patient satisfaction and enhancing their quality of life.

In this study, a significant negative relationship was found between patients’ spiritual needs and positive indicators of quality of life, such as perceived general health status and functional areas, while a positive relationship was found between spiritual needs and symptoms. It was observed that as patients’ spiritual needs increased, their quality of life decreased. The positive relationship identified between spiritual needs and symptoms experienced indicates that the needs in both areas are closely related. Spiritual distress in patients can have a significant impact on quality of life (Ferrell and Paice 2019; Taylor et al. 2014; Zumstein-Shaha et al. 2020). This study found that increased spiritual needs reduced quality of life. Supporting our research findings, Freire et al. (2014) emphasized that the physical, psychosocial, and spiritual distress experienced by cancer patients reduces quality of life and that healthcare professionals should pay more attention to this issue (Freire et al. 2014). Vallurupalli et al. determined that patients’ spirituality and religious coping are related to improved quality of life (Vallurupalli et al. 2012). Yılmaz and Cengiz (Yilmaz and Cengiz 2020) have determined that spiritual well-being contributes positively to the quality of life of cancer patients (Yilmaz and Cengiz 2020). In this study, it is thought that the increase in patients’ spiritual needs, the inability to manage existential crises, and the inability to meet the basic spiritual needs of seeking peace and religious needs negatively affect quality of life. Forouzi et al. (2017) also noted that in the relationship between spiritual needs and quality of life, spirituality can be understood as having an impact on various aspects of life through its role as a primary factor in creating meaning and expectations in life, enhancing adaptability, coping, and addressing existential crises arising from life-threatening illnesses (Forouzi et al. 2017).

This study found a positive correlation between the spiritual needs of cancer patients and their levels of depression. It was determined that as patients’ spiritual needs increased, so did the severity of their depression. Depression is a common mental health issue among cancer patients and negatively affects their quality of life (Hohls et al. 2021). It has been suggested that supporting spiritual beliefs that are meaningful to patients and their families may be effective in reducing anxiety and promoting calmness and inner peace (Zumstein-Shaha et al. 2020). Supporting our research findings, Shi et al. (2023) showed in a study with advanced cancer patients that patients’ spiritual needs were negatively associated with depression. Chen et al. (2021) found in a study with gynecological cancer patients that anxiety and depression could reduce patients’ spiritual well-being. Based on this, they stated that patients experiencing anxiety or depression require more spiritual care (Chen et al. 2021). These results confirm the previously established relationship between spirituality and psychological health (Scheffold et al. 2019; Sekely et al. 2019). Timely and adequate fulfilment of spiritual needs is critical in preventing cancer patients from falling into depression due to hopelessness. In this regard, it is necessary to provide continuous training to nurses and other healthcare professionals to develop their competence in providing spiritual care. Furthermore, the high prevalence of depression among cancer patients necessitates a strong emphasis on psychosocial and spiritual approaches in patient care, and nurses should be supported in offering interventions to protect patients from depression.

As the final finding of our study, variables predicting the spiritual needs of cancer patients were examined. The functional domain of the quality of life scale, depression level, diagnosis duration, education level, and the symptoms subdomain of the quality of life scale were determined to be independent variables predicting spiritual needs at a rate of 26.4%. It was determined that a decrease in functional domains, an increase in depression severity, an increase in diagnosis duration and education level, and an increase in symptoms affect spiritual needs. Decreases in functional areas, symptoms experienced, and depression were found to be important variables affecting spiritual needs. Similar results have been obtained in studies in the literature that address the relationship between spiritual needs, quality of life, and depression. Kurt (2015) found in his study of breast cancer patients that individuals with strong religious beliefs may have lower levels of depression and that as depression levels increase, there is a decline in quality of life, particularly in terms of functional and general health perceptions (Kurt, 2015). Chang et al. (2022) noted in their study on cancer patients that low morale in patients with oral cancer was associated with low satisfaction with spiritual needs, low quality of life, and suicidal thoughts (Chang et al. 2022). Chen et al. (2021), in their study on gynecological cancer patients, noted that religion, depression, anxiety, and quality of life were the strongest determinants of spiritual well-being. They stated that patients experiencing anxiety or depression had a greater need for spiritual care (Chen et al. 2021). Another study on breast cancer survivors examined the effects of spiritual well-being on quality of life and depression levels and found that the “meaning/peace” dimension was more decisive than the “faith” dimension in terms of psychological adjustment. They noted that the “meaning/peace” dimension made significant contributions to improving quality of life and reducing depressive symptoms (Garduño-Ortega et al. 2021).

Similarly, Simonelli et al. (2008) demonstrate that finding meaning in life effectively reduces depressive symptoms. Therefore, they argue that spiritual support centered on the individual and focused on the search for meaning should be provided. Lormans et al. (2021) recommend a spiritual and biopsychosocial approach to provide multidimensional symptom management for patients. According to this approach, providing quality spiritual care can meet patients’ spiritual needs, improve their quality of life and protect them from depression. However, when meeting these needs, it is recommended that patients are assessed individually, that care is provided with attention to their religious and cultural backgrounds, and that research is conducted in different cultures to support this outcome. The duration of diagnosis and level of education were examined as other variables affecting patients’ spiritual needs. Patients who had received their diagnosis a longer time ago and those with a university or higher education level were found to have higher spiritual needs. It is thought that a longer diagnosis duration may affect spiritual needs due to the prolonged experience of the disease and the associated feelings of hopelessness. Consistent with these findings, Sastra et al. identified a significant correlation between spiritual needs and demographic factors such as gender, age, and duration of diagnosis. They found that women and older patients had higher spiritual needs, which increased with the duration of the diagnosis (Sastra et al. 2021). It is thought that patients who are university graduates or have received an education may be better able to express their needs, which could explain this result.

### Limitation of the study

The fact that this study was conducted at a single center limits the generalizability of the results. Furthermore, the use of self-report measurement tools and the selection of patients from the universe using convenience sampling are also among the limitations of the study.

## Conclusions and recommendations

This study, which investigated the relationship between the spiritual needs of cancer patients, their quality of life, and their depression levels, found a negative relationship between increasing spiritual needs and life satisfaction, and a positive relationship with depression. Spiritual needs were found to be predicted by functional areas, depression, educational level, time since diagnosis, and symptoms. Based on this, it is possible to say that meeting the spiritual needs of cancer patients may have positive effects on improving their quality of life and reducing their depression levels.

According to the results of this study

- Nurses or health professionals should closely monitor and meet the spiritual dimension and spiritual needs of cancer patients in addition to the physical and psychological dimensions in order to provide holistic care.
- Applications that meet spiritual needs, such as spiritual counselling, worship opportunities, and religious support units, should be structured and made accessible in the care environment.
- The nursing education process should include training and practices that will provide knowledge and skills to identify and meet the spiritual needs of patients.
- In addition, future research should examine the relationship between spiritual needs and psychological variables in different disease groups and different religious and cultural contexts.
